# Allied health – 3004. Pulmonary function tests: A study of 1000 normal children

**DOI:** 10.1186/1939-4551-6-S1-P181

**Published:** 2013-04-23

**Authors:** Krishna Bihari Gupta, Krishna Bihari Gupta

**Affiliations:** 1 TB & Resp. Med, Post Graduate Institute of Medical Sciences, Rohtak, India

## Background

Respiratory infections in children have assumed epidemic proportions in recent years.Although data on pulmonary function tests of Indian children is available, very few studies in North Indian healthy children are available, especially in healthy children from Haryana. In this study, pulmonary function tests of healthy children, studying in schools of Rohtak (Haryana), on a large scale was done.

## Methods

The study was conducted on 1000 (500 boys & 500 girls) healthy, school going children of Rohtak (Haryana) of age group 10-14 years, from June 2007 to November 2008. Children having history of acute respiratory infections in preceding 3 weeks, chronic pulmonary symptoms, allergic diseases, thoracic surgery, systemic diseases which affect respiratory system and smokers were excluded from the study. Pulmonary function test was done by using portable spirometer-Spirotab I (Medical International Research Instrument ). Standard methodology for assessment of lung functions as recommended by American Thoracic Society was used. The parameters recorded in each case included forced vital capacity (FVC), forced expiratory volume in one second (FEV_1_), FEV_1_/FVC % and FEV_25-75%._Statistical analysis was done using student‘t’ test.

## Results

FEF_25-75%_showed a significant correlation with age, weight, height, body surface area and body mass index in both males and females except for correlation of FEF_25-75%_with BMI in females. In males, FVC and FEV_1_had the best correlation with body surface area followed by weight and age in both males and females. The correlation of FEV_1_/FVC% with age, weight, height, body surface area and body mass index was not significant. The mean values of all pulmonary function measurements were higher in boys as compared to girls but statistically significant difference (p<0.001) was found for FVC and FEV_1._

## Conclusions

We found positive correlation of FVC, FEV_1,_and FEF_25-75%_with age, height, BSA and BMI. There were differences in values obtained in our study and those previously conducted on Indian children, which could be attributed to other factors like, differences in body build, socioeconomic status, pollution, gender, ethnicity, etc.

